# Early Esophageal Cancer Specific Survival Is Unaffected by Anatomical Location of Tumor: A Population-Based Study

**DOI:** 10.1155/2016/6132640

**Published:** 2016-07-31

**Authors:** Rajan N. Amin, Samip J. Parikh, Venu Gopala Reddy Gangireddy, Praveen Kanneganti, Swathi Talla, Sumanth Daram

**Affiliations:** ^1^University of South Carolina School of Medicine, Columbia, SC, USA; ^2^University Hospital, Augusta, GA, USA; ^3^Division of Gastroenterology and Hepatology, Georgia Regents University, Augusta, GA, USA; ^4^Baptist Memorial Hospital, Memphis, TN, USA

## Abstract

*Background*. Approximately one-fifth of all esophageal cancer cases are defined as early esophageal cancer (EEC). Although endoscopic therapy (ET) has been shown to be equally effective as esophagectomy (EST) in patients with EEC, there is little information comparing the survival outcomes of the two therapies based on anatomical location.* Methods*. A population-based study was conducted and the data was obtained from Surveillance, Epidemiology, and End Results program. Patients with EEC (i.e., stages Tis and T1a) and treated with either ET or EST were analyzed to compare EEC-related survival for three different locations of tumor.* Results*. The overall EEC-specific 1-year and 5-year mean (±SE) survival rates were 11.66 ± 0.05 and 52.80 ± 0.58 months, respectively. Tumors located in lower third had better 5-year survival compared to those located in middle third (83.50% versus 73.10%, *p* < 0.01). However, when adjusted for age, race, gender, marital status, grade, stage of tumor, histological type, and treatment modality, there was no significant difference.* Conclusion*. The EEC-specific 1-year or 5-year adjusted survival did not differ by anatomic location of the tumor. Therefore, ET might serve as a minimally invasive yet effective alternative to EST to treat EEC.

## 1. Introduction

Esophageal cancer is one of the most aggressive cancers in the world. It is the eighth most common cause of cancer globally, with an estimated 456,000 newly diagnosed cases in 2012 (3.2% of total cancers), and the sixth leading cause of cancer death with an estimated 400,000 deaths (4.9% of total cancers). It is more common in developing countries in Asia and Africa, with lower incidence in Europe and North America [1]. The incidence of esophageal cancer continues to increase in the United States, with an estimated 16,980 new cases and 15,590 deaths attributed to this cancer in 2015 [2]. The overall 5-year survival is 17.9%. The stage of the disease has significant impact on the overall survival. The 5-year survival for localized cancers is 40.4%; regional cancer is 21.6%; and distant metastatic cancer is 4.2% [3]. Approximately one-fifth of all esophageal cancer cases are defined as early esophageal cancer (EEC), which is limited to the intraepithelial (Tis), mucosa (T1a), and/or submucosa (T1b) cancers. Tumors that have not penetrated the muscularis mucosa (stage T1a) are rarely accompanied by lymph node metastasis (0–2%), which makes these lesions suitable for endoscopic resection [4, 5].

Although associated with high rates of morbidity and mortality, esophagectomy (EST) has served as the treatment of choice for EEC. However, endoscopic therapy (ET) has gradually gained acceptance for the treatment of EEC, especially for adenocarcinomas arising from Barrett's esophagus. Studies have shown that the two therapies are equally effective in patients with EEC [4–9].

Several studies have indicated that location of the tumor affects survival rates for esophageal cancer [10–14]. Although data suggests that the overall survival of EEC patients treated with ET is comparable to those treated with EST, there is little information comparing the outcomes of the two therapies based on anatomical location.

The primary objective of our study was to determine whether anatomical location of the tumor affects overall outcomes in patients with EEC. We hypothesized that there is no difference in 1-year or 5-year survival with respect to anatomical location of tumor. The secondary objectives were (1) to compare overall EEC-specific 1-year and 5-year survival with respect to treatment therapy, histological grade, and staging of tumors; (2) to compare survival outcome between ET and EST with respect to location of tumor; and (3) to evaluate the predictors of EEC-specific mortality.

## 2. Patients and Methods

### 2.1. Data Source and Study Population

The data was collected from the Surveillance Epidemiology and End Results (SEER) program of the National Cancer Institute (released in 2015) [15]. SEER collects cancer-related survival data and related variables from 18 regional registries in the US that cover approximately 27.8% of the US population (based on 2010 census). The 18 SEER registries are Alaska Native Tumor Registry, Arizona Indians, Cherokee Nation, Connecticut, Detroit, Georgia Center for Cancer Statistics (Atlanta, Greater Georgia, and Rural Georgia), Greater Bay Area Cancer Registry (San Francisco-Oakland and San Jose-Monterey), Greater California, Hawaii, Iowa, Kentucky, Los Angeles, Louisiana, New Jersey, New Mexico, Seattle-Puget Sound, and Utah. All the data that was collected by SEER is deidentified; hence this study was exempt from institutional review board review (Office of Human Subject Research; National Institutes of Health).

We identified all patients with primary esophageal tumors localized to upper, mid, and lower esophagus from 1998 to 2012. Only cases with known age, microscopically confirmed with active follow-up, known stage, and definitive therapy (ET or EST), were included in the study. We defined early esophageal cancer (EEC) as tumor involving the intraepithelial (Tis) and mucosal (T1a) regions only. All cases diagnosed with death certificate or autopsy only, alive with no survival times, and deaths due to other causes with no survival time were excluded from the study. Cases with age values not found, invalid year, and values not found for other variables were also excluded to match the expected survival table.

The primary site and morphology codes C15.0 and C15.3 were used to identify tumors localized to upper esophagus, C15.4 was used to identify mid esophagus, and C15.2 and C15.5 were used to identify lower esophagus. CS extension (2004+) and EOD 10 extent (1988–2003) were used to define the stage of the cancer. Stage Tis (carcinoma in situ) was defined as noninvasive and intraepithelial tumor. Stage T1a was defined as tumor involving lamina propria or muscularis mucosa. Histologic recode broad groupings were used to define the nature of the tumor. Codes 8140–8389 were used to define adenomas and adenocarcinomas; codes 8050–8089 for squamous cell neoplasms; and all other remaining codes as other histology. Surgery codes 10–27 were used to define ET and codes 30–80 were used to define EST. The surgical treatments include partial esophagectomy, total esophagectomy, and esophagectomy (with laryngectomy and/or gastrectomy, partial gastrectomy, or total gastrectomy). The endoscopic treatments include local tumor destruction (photodynamic therapy, electrocautery, cryosurgery, or laser) and local tumor excision (polypectomy or excisional biopsy). Covariates that were included for the study were age, gender, race, marital status, size of the tumor, histology, and grade of the tumor.

### 2.2. Statistical Analysis

EEC-specific survival outcomes for all patients were determined using SEER data from 1998 to 2012. SEER Stat software (version 8.2.1) was used to obtain all case listings. End calculated vital status was used to determine EEC-specific 1-year and 5-year endpoint (dead or alive).

Chi-square and one-way ANOVA tests were used to compare categorical and continuous variables of patient characteristics among three groups based on tumor location. Kaplan-Meier univariate survival curves and log-rank test were used to compare 1-year and 5-year EEC-related survival for three different locations of tumor. Multivariate Cox regression analyses were performed to adjust for the concurrent effect of multiple variables on the survival. The covariates in the prediction model were determined based on clinical relevance and univariate analysis. Two sided *p* value < 0.05 was considered significant. We performed all statistical analyses with SPSS software version 22.0 (IBM Corp, Armonk, NY).

## 3. Results

### 3.1. General Characteristics

A total of 1330 patients [mean (±SE) age: 64.75 ± 0.29 years; 83.0% males; 93.7% whites] with microscopically confirmed stage Tis (20.3%) and stage T1a (79.8%) between 1998 and 2012 were identified. The largest proportion of tumors was located in lower third (82.7%), followed by middle third (13.2%) and upper third (4.1%) of the esophagus. The adenocarcinomas are the most common esophageal cancers constituting about 48%, 50%, and 81% of upper, mid, and lower esophagus, respectively. About two-thirds (66.8%) of the patients underwent EST, while the remaining (33.2%) had ET.  Table 1 summarizes the demographic and cancer-related variables of these patients by anatomical location of the tumor.

### 3.2. Overall EEC-Specific 1-Year and 5-Year Survival

Overall, estimated EEC-specific 1-year and 5-year mean (±SE) survival were 11.66 ± 0.05 and 52.80 ± 0.58 months, respectively. Tables 2 and 3 illustrate EEC-specific 1-year and 5-year survival percentages with respect to treatment modality, histological grade, location, and staging of tumors, respectively. There was a significant difference in EEC-specific 5-year survival, but not with 1-year survival, with respect to the location of the tumor. In a pairwise comparison, tumors located in lower third had better 5-year survival percentages compared to those located in middle third (83.50% versus 73.10%, *p* < 0.01). Tumors with stage T1a had significantly lower EEC-specific 5-year survival compared with those with stage Tis. Histologically, patients with adenocarcinoma were found to have better 1-year (94.90% versus 90.50%, *p* = 0.03) and 5-year (83.40% versus 67.90%, *p* < 0.01) survival compared to those with squamous cell carcinoma. EEC-specific 1-year and 5-year survival were not different between ET and EST.

### 3.3. Survival Outcome between ET and EST with respect to Location of Tumor

**Figure 1:**
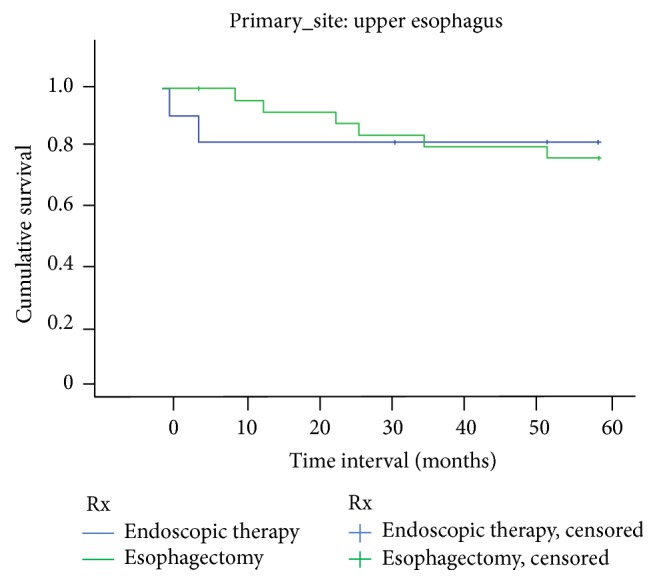
Upper one-third EEC-related survival based on treatment.

**Figure 2:**
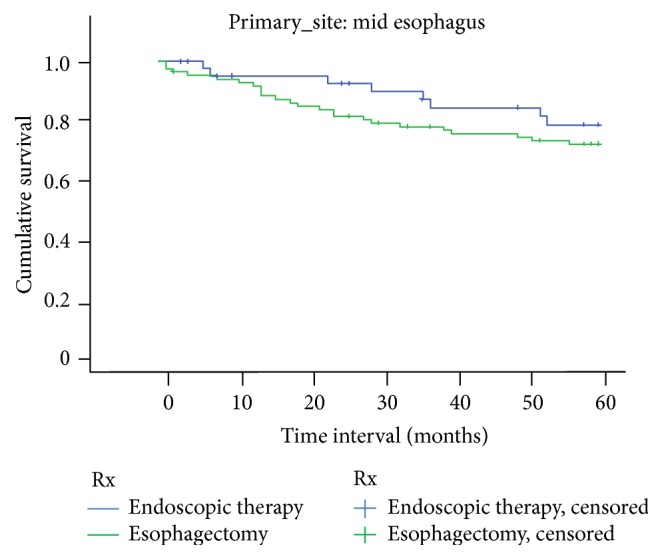
Middle one-third EEC-related survival based on treatment.

**Figure 3:**
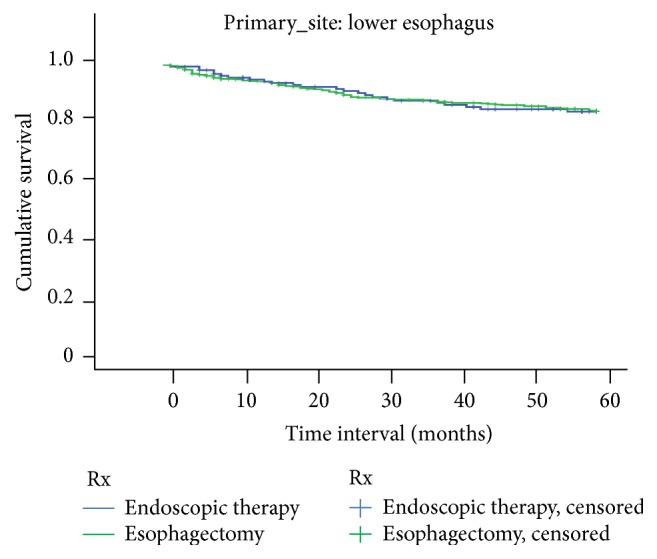
Lower one-third EEC-related survival based on treatment.

There was no significant difference in survival between ET and EST at both 1 year and 5 years for tumors localized to upper third of the esophagus.  Figure 1 shows the Kaplan-Meier survival curve for upper esophagus. Similar results were noted for tumors located in the middle and lower third of the esophagus. Figures 2 and 3 show Kaplan-Meier curves for middle and lower esophagus.  Table 4 summarizes the 1-year and 5-year EEC-specific survival outcomes between ET and EST based on location of tumor.

### 3.4. Predictors of EEC-Specific Mortality

The results of the multivariate Cox proportion hazard regression analyses in Table 5 revealed no association of 1-year and 5-year EEC-specific mortality with location of tumor, age, race, sex, marital status, size, grade, stage of tumor, histological type, and treatment (all *p* > 0.05). Patients with “undifferentiated or grade IV” tumors had 4.12 times higher chance of dying at 5 years as compared to those with “well-differentiated or grade I” tumors (CI 1.09–15.62, *p* = 0.04).

## 4. Discussion

In this population-based study, we found that there is no EEC-specific 1-year survival difference with respect to location of the tumor. There appears to be EEC-specific 5-year survival benefits for tumors located in the lower third compared to middle third of the esophagus. However, when adjusted for age, race, sex, marital status, size, grade, stage of tumor, histological type, and treatment modality, there was no significant difference. In addition, the treatment modality (ET or EST) did not affect 1-year or 5-year survival with respect to location of the tumor.

Esophagectomy has served as the standard treatment for EEC patients due to high tumor-free survival rates. However, this procedure is associated with a mortality rate of about 1-2% at high volume centers and 5–10% at low volume centers [16]. This procedure is also associated with a morbidity rate of 30–50% [17]. In one study EST was associated with major and minor complication rates of about 13% and 63%, respectively. Complications of EST include anastomotic leaks, anastomotic strictures, and atrial fibrillation [9]. These adverse events have led to a growing interest in ET. Recent studies have indicated that ET provides favorable long-term results [18–20]. Several large studies have also shown that ET has comparable survival rates to EST, making it a viable alternative in the treatment of EEC [4–9]. Additionally, previous studies have shown that location of tumor does not affect the survival in patients treated with EST [12–14].

Previous studies have compared survival outcomes between EST and ET for the treatment of EEC [4–9]. Wani et al. found no differences in the 2-year and 5-year survival rate in EEC between the EST and ET groups [5]. Das et al. also found similar results when comparing EST with ET in patients with EEC [4]. Another recent SEER based analysis comparing EST with ET in an older population (age ≥ 66 years) with EEC (T0 and T1a) showed improved short term (60 days) and long-term (2 years) outcomes in ET group despite being older and having more comorbidities than EST group [21]. The above two studies have similar strengths and weaknesses compared to our present study given that the use of the SEER database was common. The key difference between our study and the earlier studies is that we compared survival rates with the two therapies with respect to tumor location within the esophagus. Additional strength of our study is that there were no cases lost to follow-up. The exact survival outcomes (i.e., alive or dead) were known for all the cases included in the study. In a single center study, Prasad et al. compared outcomes between EST and ET and found similar survival rates between the two groups [8].

We did not observe any 1-year or 5-year EEC-specific survival benefits with respect to location of the tumor. A few studies suggest that tumor location within the esophagus impacts long-term survival [10, 11]. In one study, Eloubeidi et al. used the SEER database to determine prognostic factors for esophageal cancer survival and found that tumors located in the lower esophagus were associated with increased mortality [10]. In contrast, a study by Li et al. showed that squamous cell carcinomas located in the lower esophagus had better prognosis compared to other sites, possibly due to better surgical outcomes [11]. However, other studies indicate that the location of the tumor does not affect survival [12–14]. Otterstatter et al. found that the 5-year survival rates for esophageal cancer in Canada were similar regardless of whether the tumor was located in the upper, middle, or lower esophagus [13]. Doki et al. looked at 501 patients with primarily squamous cell carcinoma and found similar 5-year survival rates between the three sites [12]. However, they found that tumor location impacted the mode of tumor recurrence.

One of the advantages of the SEER database is that it is a large population-based database that includes information from multiple academic and community based institutions from around the country. This provides greater generalizability of results and allows for specific subgroups of cancers to be compared. The information collected by this database undergoes rigorous data collection procedures and quality control standards, which ensures highly accurate and reliable data.

There are some limitations to our study. The SEER database does not report data on local recurrence. However, the comparable survival rates indicate that this lack of information on incomplete resection and local recurrence should not influence our overall conclusions. SEER only reports first treatment interventions in the patients. Hence recurrence of esophageal cancer and subsequent treatments were not known. Another limitation of this study is that information on comorbidities and socioeconomic factors such as smoking and alcohol use was not collected in the SEER database. It is possible that patients with more significant comorbidities were selected for ET, which could in turn have led to a decrease in magnitude of favorable outcomes with ET. Our study tried to limit this bias by focusing on EEC-specific survival rather than crude survival statistics. Also, the exact etiology of the deaths, procedure (ET and EST) related complications, and recurrence rates were not reported in SEER database.

In conclusion, the results from this population-based study demonstrate that EEC-specific adjusted 1-year or 5-year survival did not differ by location of the tumors. Moreover, EEC patients treated with ET or EST exhibit comparable 1-year and 5-year survival with respect to anatomical location of tumor. Therefore, ET might serve as a minimally invasive yet effective alternative to EST to treat EEC. Further prospective randomized clinical trials are needed to validate these findings.

## Figures and Tables

**Table 1:** General characteristics of patients based on location of tumor.

Characteristic	Total	Upper	Middle	Lower	*p* value
*Total*	*N* = *1330 (100%)*	*N* = *54 (4.1%)*	*N* = *176 (13.2%)*	*N* = *1100 (82.7%)*	

*Mean ± SE age (year)*	64.75 ± 0.29	64.54 ± 1.42	65.69 ± 0.82	64.61 ± 0.31	0.43

*Sex (% males)*	1104 (83.0%)	32 (59.3%)	130 (73.9%)	942 (85.6%)	<0.01

*Race (% Whites)*	1246 (93.7%)	45 (83.3%)	149 (84.7%)	1052 (95.6%)	<0.01

*Histology*					
Squamous cell carcinoma	158 (11.9%)	22 (40.7%)	72 (40.9%)	64 (5.8%)	<0.01
Adenocarcinoma	1011 (76.0%)	26 (48.1%)	89 (50.6%)	896 (81.5%)	
Others	161 (12.1%)	6 (11.1%)	15 (8.5%)	140 (12.7%)	

*Tumor stage*					
Stage Tis	270 (20.3%)	17 (31.5%)	40 (22.7%)	213 (19.4%)	0.07
Stage T1a	1060 (79.8%)	37 (68.5%)	136 (77.3%)	887 (80.0%)	

*Tumor grade*					
Well-differentiated	169 (12.7%)	9 (16.7%)	21 (11.9%)	139 (12.6%)	0.17
Moderately differentiated	363 (27.3%)	8 (14.8%)	49 (27.8%)	306 (27.8%)	
Poorly differentiated	175 (13.2%)	10 (18.5%)	31 (17.6%)	134 (12.2%)	
Anaplastic	16 (1.2%)	1 (1.9%)	0 (0%)	15 (1.4%)	
Unknown	607 (45.6%)	26 (48.1%)	75 (42.6%)	506 (46.0%)	

*Tumor size*					
No mass (0 mm)	1 (0.1%)	0 (0%)	0 (0%)	1 (0.2%)	0.48
Microscopic focus only (1 mm)	58 (8.5%)	5 (17.2%)	5 (5.0%)	48 (8.7%)	
2 mm–990+ mm (up to 997 mm)	622 (90.9%)	24 (82.8%)	96 (95.0%)	502 (90.6%)	
Entire circumference (998 mm)	3 (0.4%)	0 (0%)	0 (0%)	3 (0.5%)	

*Treatment modality*					
Endoscopic treatment	411 (30.9%)	25 (46.3%)	65 (36.9%)	321 (29.2%)	<0.01
Esophagectomy	919 (69.1%)	29 (53.7%)	111 (63.1%)	779 (70.8%)	

**Table 2:** EEC-specific 1-year survival percentage.

Characteristic	*N*	Number of deaths	1-year % survival	Mean 1-year survival (95% CI), months	*p* value
*Overall survival*	1330	71	94.70%	11.66 (11.57–11.75)	

*Treatment*					
Endoscopic treatment	411	18	95.60%	11.74 (11.60–11.88)	0.30
Esophagectomy	919	53	94.20%	11.63 (11.52–11.74)	

*Histological grade*					
Adenocarcinoma	1011	52	94.90%	11.65 (11.54–11.75)	0.02
Squamous cell carcinoma	158	15	90.50%	11.52 (11.24–11.80)	
Others	161	4	97.50%	11.88 (11.76–12.00)	

*Location*					
Upper	54	4	92.60%	11.43 (10.82–12.03)	0.48
Middle	176	12	93.20%	11.60 (11.33–11.86)	
Lower	1100	55	95.00%	11.68 (11.59–11.78)	

*Staging*					
Stage Tis	270	12	95.60%	11.69 (11.50–11.89)	0.48
Stage T1a	1060	59	94.40%	11.65 (11.55–11.75)	

**Table 3:** EEC-specific 5-year survival percentage.

Characteristics	*N*	Number of deaths	5-year % survival	5-year survival (95% CI), months	*p* value
*Overall survival*	848	155	81.70%	52.80 (51.68–53.93)	

*Treatment*					
Endoscopic treatment	201	34	83.10%	53.18 (50.93–55.43)	0.81
Esophagectomy	647	121	81.30%	52.69 (51.38–53.99)	

*Histological grade*					
Adenocarcinoma	619	103	83.40%	53.36 (52.07–54.65)	<0.01
Squamous cell carcinoma	112	36	67.90%	47.47 (43.73–51.21)	
Others	117	16	86.30%	55.18 (52.81–57.54)	

*Location*					
Upper	34	8	76.50%	50.66 (44.43–56.89)	0.02
Middle	119	32	73.10%	49.84 (46.49–53.18)	
Lower	695	115	83.50%	53.42 (52.21–54.63)	

*Staging*					
Stage Tis	205	20	90.20%	55.84 (53.97–57.70)	<0.01
Stage T1a	643	135	79.00%	51.83 (50.47–53.93)	

**Table 4:** EEC-related survivals between two treatment groups based on location of tumor.

Location of tumor	Total number	1-year survival percentage	5-year survival (95% CI), months	*p* value	Total number	5-year survival percentage	5-year survival (95% CI), months	*p* value
*Upper*								
ET	25	88.00%	10.84 (9.58–12.10)	0.23	10	80.00%	48.60 (34.46–62.74)	0.87
EST	29	96.60%	11.93 (11.79–12.07)		24	75.00%	51.48 (44.90–58.06)	

*Middle*								
ET	65	95.40%	11.81 (11.57–12.05)	0.39	40	80.00%	53.21 (48.44–57.98)	0.34
EST	111	91.90%	11.48 (11.08–11.87)		79	69.60%	48.27 (43.94–52.60)	

*Lower*								
ET	321	96.30%	11.79 (11.65–11.93)	0.21	151	84.10%	53.46 (50.91–56.00)	0.99
EST	779	94.50%	11.64 (11.52–11.76)		544	83.30%	53.40 (52.03–54.78)	

**Table 5:** Multivariate Cox proportion hazard regression analyses.

Variables	1-year (*N* = 1330)		5-year (*N* = 848)	
	HR (95% CI)	*p* value	HR (95% CI)	*p* value
*Age at diagnosis*	1.01 (0.98–1.05)	0.53	0.99 (0.97–1.02)	0.53

*Sex*				
Females	Reference		Reference	
Males	1.07 (0.45–2.55)	0.53	0.79 (0.46–1.36)	0.40

*Marital status*				
Single (never married)	Reference		Reference	
Married (including common law)	2.20 (0.64–7.63)	0.33	1.48 (0.68–3.22)	0.33
Divorced/separated/widowed/unmarried or domestic partner	2.35 (0.59–9.32)	0.23	1.31 (0.54–3.17)	0.55
Unknown	0.00 (0.00–3.48)	0.78	0.48 (0.06–3.99)	0.50

*Race*				
Caucasians	Reference		Reference	
African Americans	1.81 (0.49–6.78)	0.38	1.32 (0.54–3.17)	0.55
Others	2.17 (0.64–7.38)	0.75	0.78 (1.72–3.53)	0.75
Unknown	0.00 (0.00–2.05)	0.93	0.00 (0.00–5.06)	0.91

*Treatment*				
ET	Reference		Reference	
EST	0.64 (0.30–1.37)	0.25	0.71 (0.35–1.44)	0.34

*Location of tumor*				
Lower	Reference		Reference	
Middle	1.39 (0.57–3.37)	0.47	1.26 (0.67–2.39)	0.47
Upper	0.38 (0.05–3.02)	0.36	1.28 (0.52–3.15)	0.59

*Grade of tumor*				
Well-differentiated; grade I	Reference		Reference	
Moderately differentiated; grade II	1.66 (0.61–4.52)	0.33	1.30 (0.62–2.75)	0.49
Poorly differentiated; grade III	1.84 (0.60–5.68)	0.29	1.76 (0.82–3.75)	0.15
Undifferentiated; anaplastic; grade IV	5.27 (0.92–30.21)	0.06	4.12 (1.09–15.62)	0.04
B-cell precursor/unknown	0.58 (0.18–1.90)	0.37	0.77 (0.34–1.74)	0.52

*Size of tumor*				
Entire circumference	Reference		Reference	
Microscopic focus only	0.37 (0.04–3.55)	0.39	0.73 (0.09–6.08)	0.77
2 mm to 990+ mm	0.17 (0.02–1.33)	0.09	0.45 (0.06–3.33)	0.43
No mass	0.00 (0.00–0.00)	0.99	0.00 (0.00–3.96)	0.97

*Stage of tumor*				
T1a	Reference		Reference	
Tis	2.27 (0.73–7.00)	0.93	1.04 (0.45–2.42)	0.93

*Histology of tumor*				
Adenocarcinoma	Reference		Reference	
Squamous cell carcinoma	1.62 (0.61–4.34)	0.34	1.68 (0.86–3.28)	0.13
Others	0.29 (0.04–2.17)	0.23	0.82 (0.36–1.85)	0.62

HR: hazard ratio; CI: confidence interval; EET: endoscopic therapy; EST: esophagectomy.
